# Supplementary figures and images for: Sox2 and FGF20 interact to regulate organ of Corti hair cell and supporting cell development in a spatially-graded manner
Source: PLoS Genet. 2019 Jul 5;15(7):e1008254. doi: 10.1371/journal.pgen.1008254 (PMC6636783; doi:10.1371/journal.pgen.1008254)

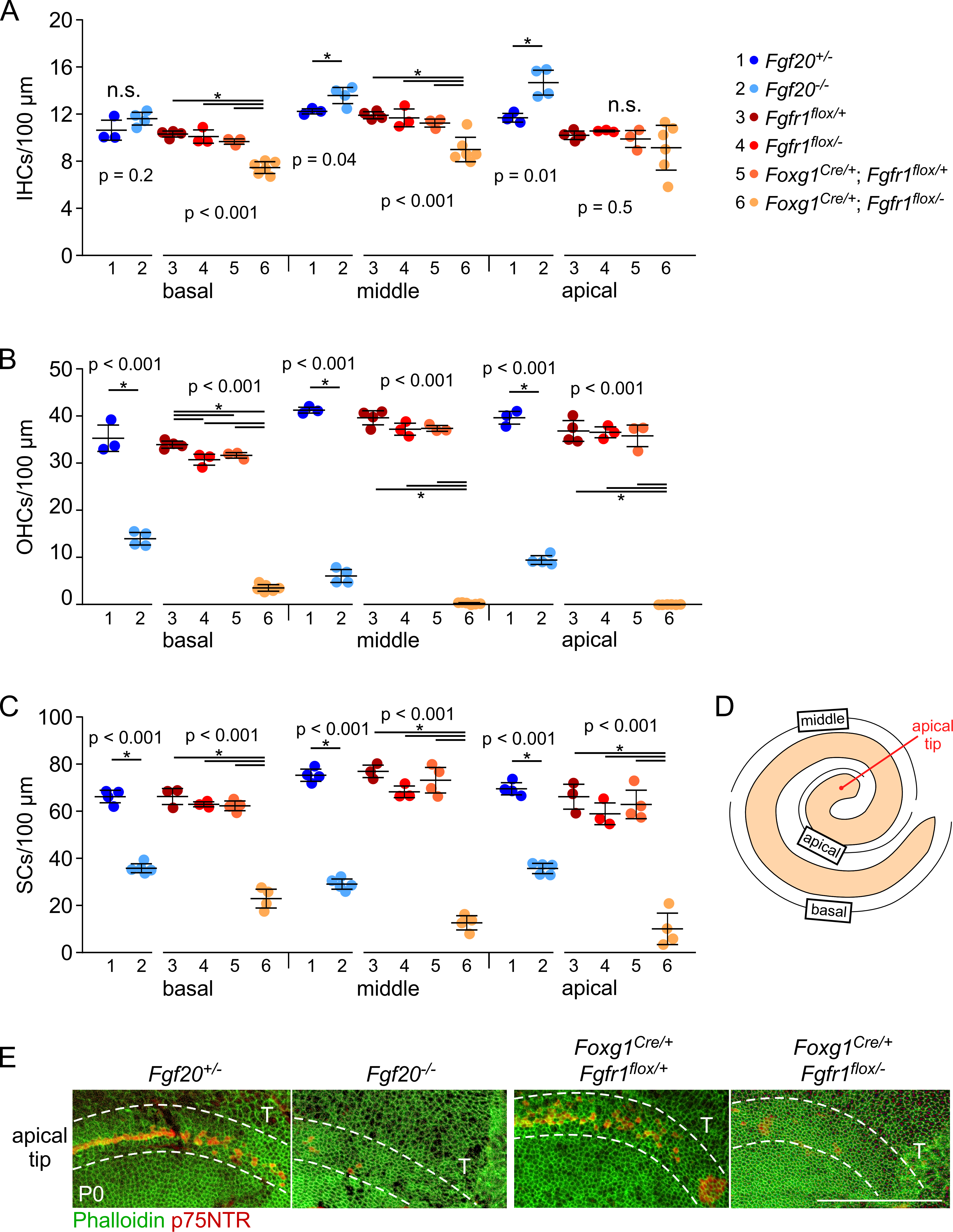

Supplement: S1 Fig — (A-C) Quantification of length-normalized number of (A) inner hair cells (IHCs/100 μm), (B) outer hair cells (OHCs/100 μm), and (C) supporting cells (SCs/100 μm) in the basal, middle, and apical turns of P0 cochleae from Fgf20+/-, Fgf20-/-, and Fgfr1flox/+, Fgfr1flox/-, Foxg1Cre/+;Fgfr1flox/+, and Foxg1Cre/+;Fgfr1flox/- mice. Fgf20+/- and Fgf20-/- cochleae were analyzed by unpaired Student’s t test; Fgfr1flox/+, Fgfr1flox/-, Foxg1Cre/+;Fgfr1flox/+, and Foxg1Cre/+;Fgfr1flox/- cochleae were analyzed by one-way ANOVA. P values shown are from the t test and ANOVA. * indicates p < 0.05 from Student’s t test or Tukey’s HSD (ANOVA post-hoc); n.s., not significant. Error bars, mean ± SD. n = (A, B) 3, 4, 4, 3, 3, 6; (C) 4, 5, 3, 3, 4, 4. (D) Schematic showing the positions of basal, middle, and apical turns along the cochlear duct. Apical tip refers to the apical end of the cochlea. (E) Whole mount cochlea from P0 Fgf20+/-, Fgf20-/-, and Foxg1Cre/+;Fgfr1flox/+, Foxg1Cre/+;Fgfr1flox/- mice showing immunofluorescence for phalloidin (green) and p75NTR (red) at the apical tip of the cochlea. T, towards the tip. Scale bar, 100 μm. Samples are representative of n = 3, 4, 3, 6. (TIF) [file pgen.1008254.s001.tif]

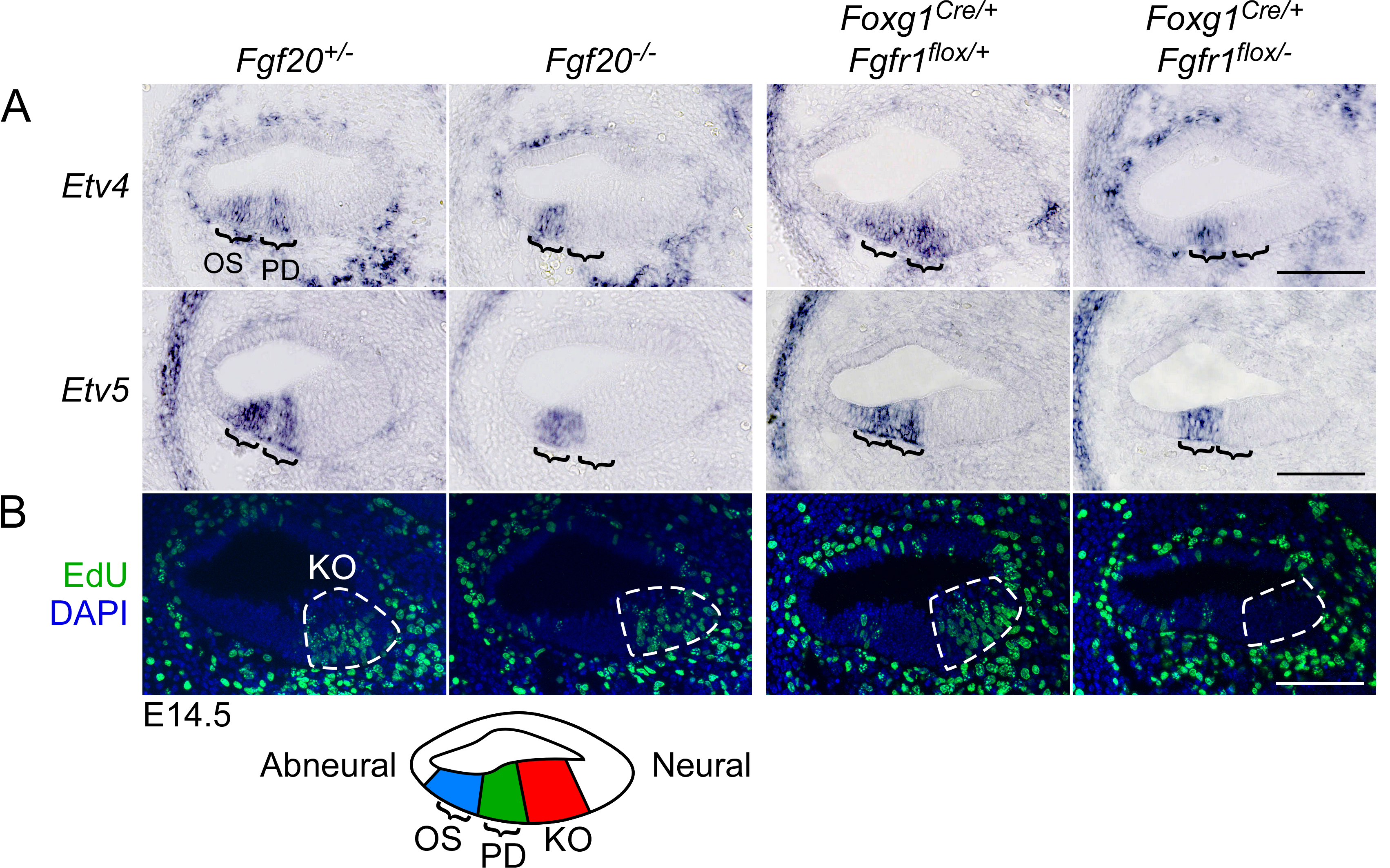

Supplement: S2 Fig — (A, B) Sections through the middle turn of E14.5 cochlear ducts from Fgf20+/-, Fgf20-/-, and Foxg1Cre/+;Fgfr1flox/+, Foxg1Cre/+;Fgfr1flox/- mice. Scale bar, 100 μm. Refer to schematic below. OS, outer sulcus; PD, prosensory domain; KO, Kölliker’s organ. (A) RNA in situ hybridization for Etv4 and Etv5. The two brackets indicate Etv4/5 expression in the outer sulcus (OS, left) and prosensory domain (PD, right; lost in Fgf20-/- and Foxg1Cre/+;Fgfr1flox/- cochleae). Samples are representative of n = (Etv4) 3, 3, 4, 4; (Etv5) 3, 3, 4, 4. (B) EdU-incorporation (green). Dashed region indicates Kölliker’s organ (KO). DAPI, nuclei (blue). Samples are representative of n = 3, 3, 3, 3. (TIF) [file pgen.1008254.s002.tif]

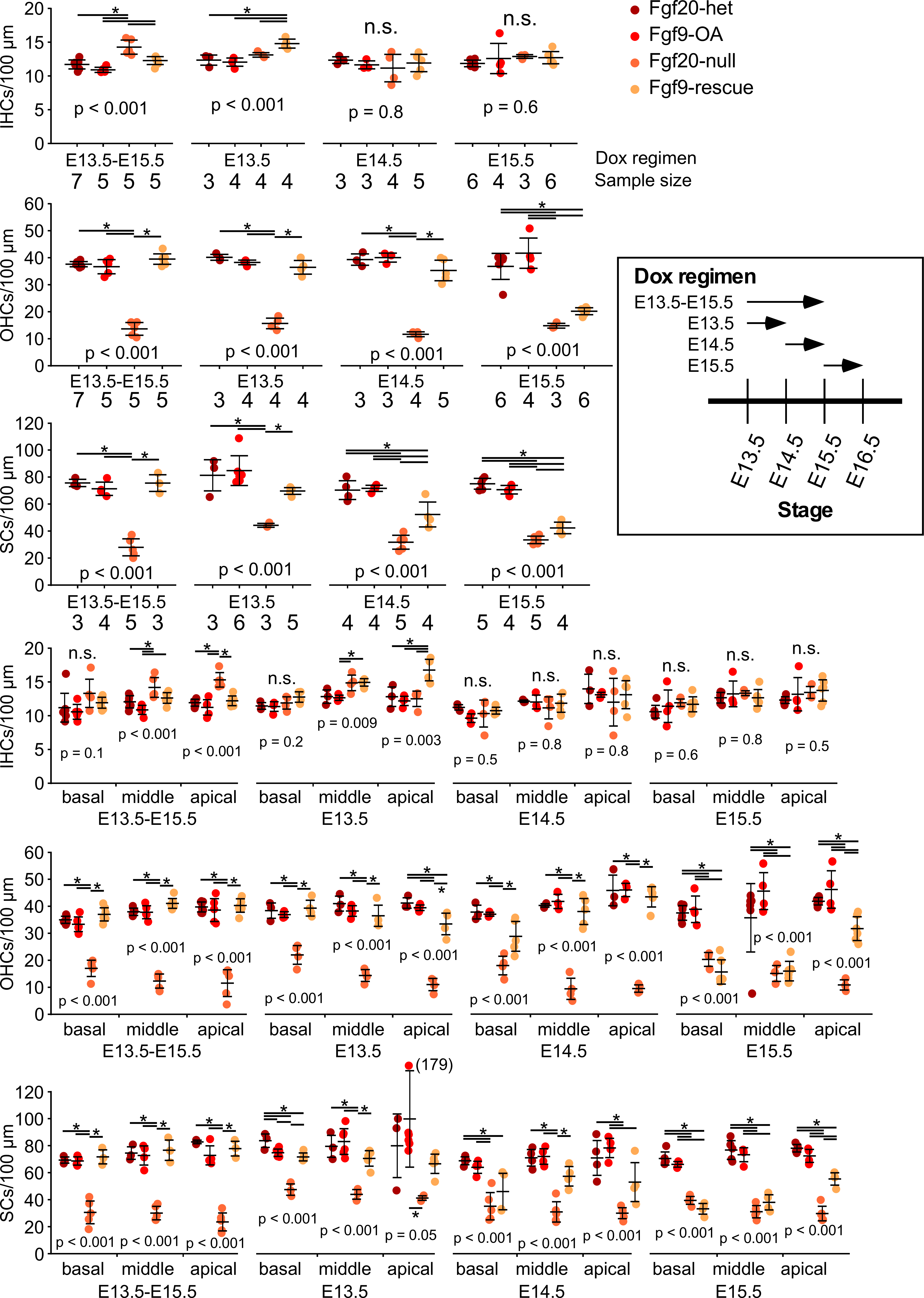

Supplement: S3 Fig — Quantification of length-normalized number of inner hair cells (IHCs/100 μm), outer hair cells (OHCs/100 μm), and supporting cells (SCs/100 μm) overall (along the entire cochlea; top three graphs) and in the basal, middle, and apical turns (bottom three graphs) of P0 cochleae from Fgf20+/-;ROSArtTA (Fgf20-het), Fgf20+/-;ROSArtTA; TRE-Fgf9-IRES-eGfp (Fgf9-OA), Fgf20-/-;ROSArtTA (Fgf20-null), and Fgf20-/-;ROSArtTA; TRE-Fgf9-IRES-eGfp (Fgf9-rescue) mice. Dox regimens: E13.5-E15.5, E13.5, E14.5, or E15.5 (see box for schematic showing the Dox diet start and stop times for each regimen). P values shown are from one-way ANOVA. * indicates p < 0.05 from Tukey’s HSD (ANOVA post-hoc); n.s., not significant. Error bars, mean ± SD. Summarized in Fig 3C. Sample sizes are indicated below the graphs. (TIF) [file pgen.1008254.s003.tif]

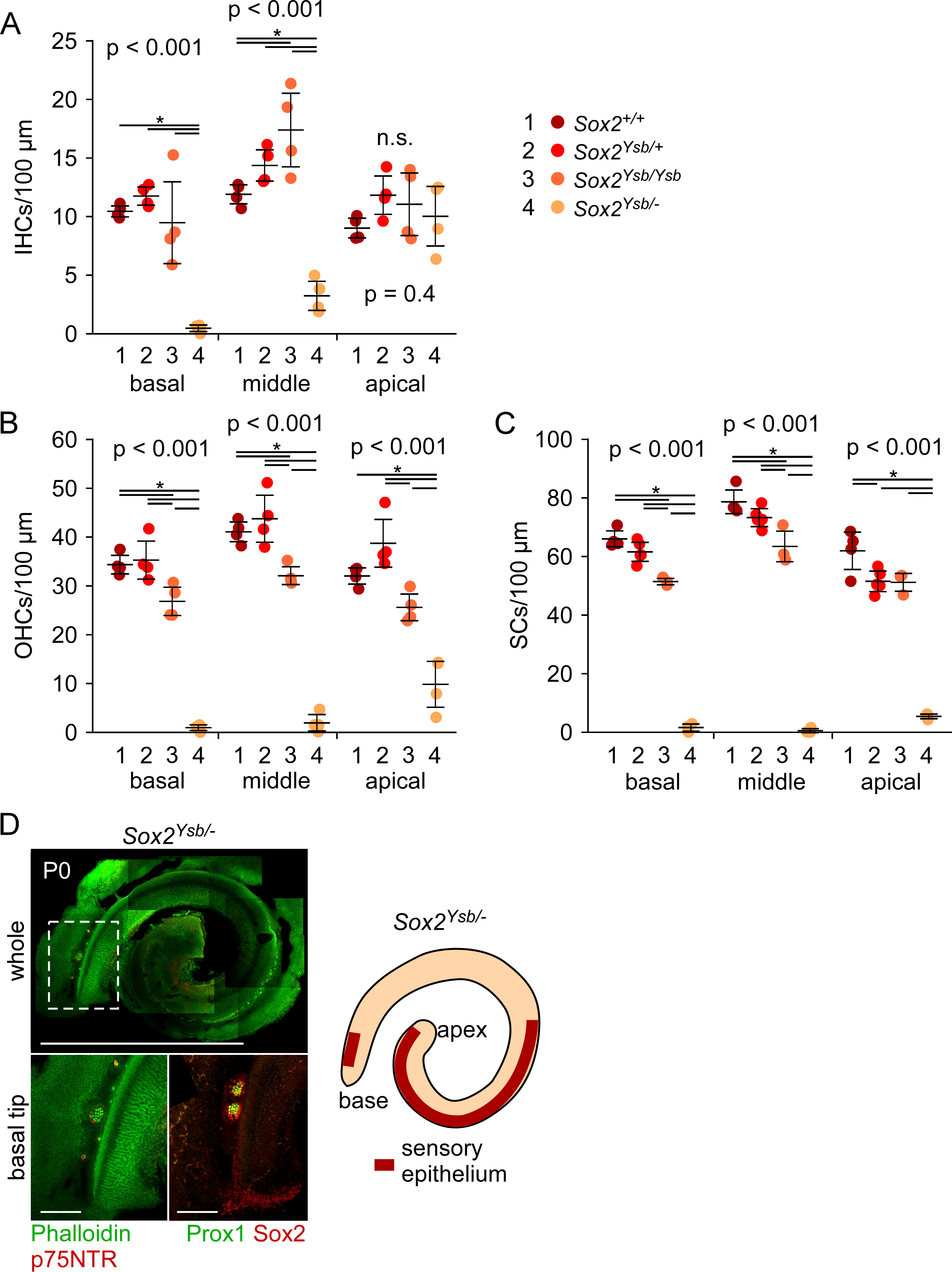

Supplement: S4 Fig — (A-C) Quantification of length-normalized number of (A) inner hair cells (IHCs/100 μm), (B) outer hair cells (OHCs/100 μm), and (C) supporting cells (SCs/100 μm) in the basal, middle, and apical turns of P0 cochleae from Sox2+/+, Sox2Ysb/+, Sox2Ysb/Ysb, and Sox2Ysb/- mice. P values shown are from one-way ANOVA. * indicates p < 0.05 from Tukey’s HSD (ANOVA post-hoc); n.s., not significant. Error bars, mean ± SD. n = (A, B) 4, 4, 4, 4; (C) 4, 5, 3, 3. (D) Whole mount cochlea from P0 Sox2Ysb/- mice showing presence of inner and outer hair cells (phalloidin/p75NTR) and supporting cells (Prox1/Sox2, in a different cochlea) at the basal tip. Schematic shows the location of sensory epithelium at the apical turn and basal tip of Sox2Ysb/- cochleae. Scale bar, 1 mm (whole), 100 μm (basal tip). (TIF) [file pgen.1008254.s004.tif]

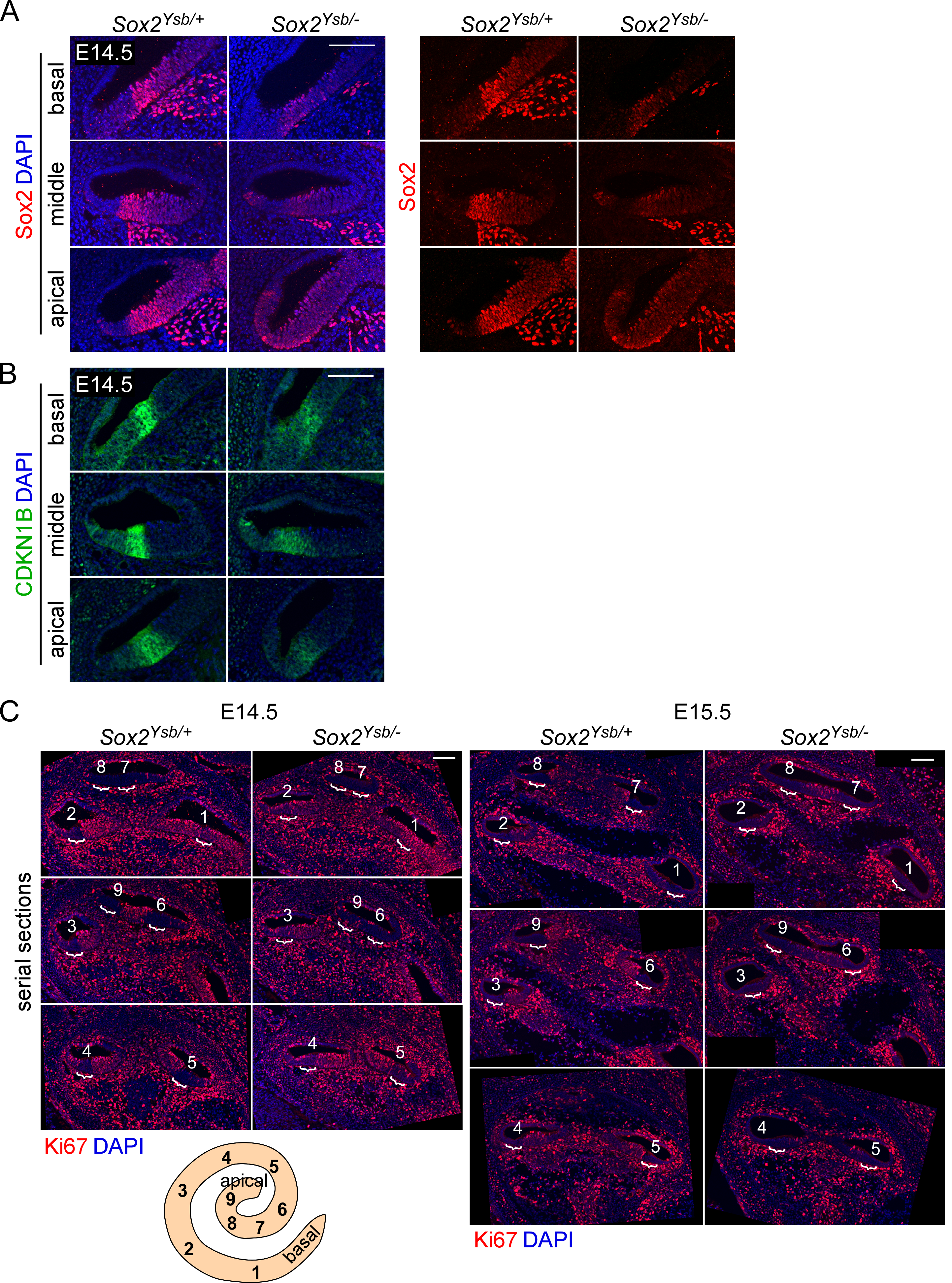

Supplement: S5 Fig — (A, B) Immunofluorescence for (A) Sox2 (red) and (B) CKDN1B (green) in sections through the basal, middle, and apical turns of E14.5 Sox2Ysb/+ and Sox2Ysb/- cochleae. Samples are representative of n = (A) 5, 6; (B) 3, 3. (C) Immunofluorescence for Ki67 (red) on serial “mid-modiolar” sections through the E14.5 and E15.5 Sox2Ysb/+ and Sox2Ysb/- cochleae. Brackets indicate prosensory domain. Nine sections through the length of the cochlear duct are labeled. See whole mount cochlear duct schematic (lower left) for relative positions of the sections. Samples are representative of n = (E14.5) 3, 3; (E15.5) 3, 3. DAPI, nuclei (blue). Scale bar, 100 μm. (TIF) [file pgen.1008254.s005.tif]

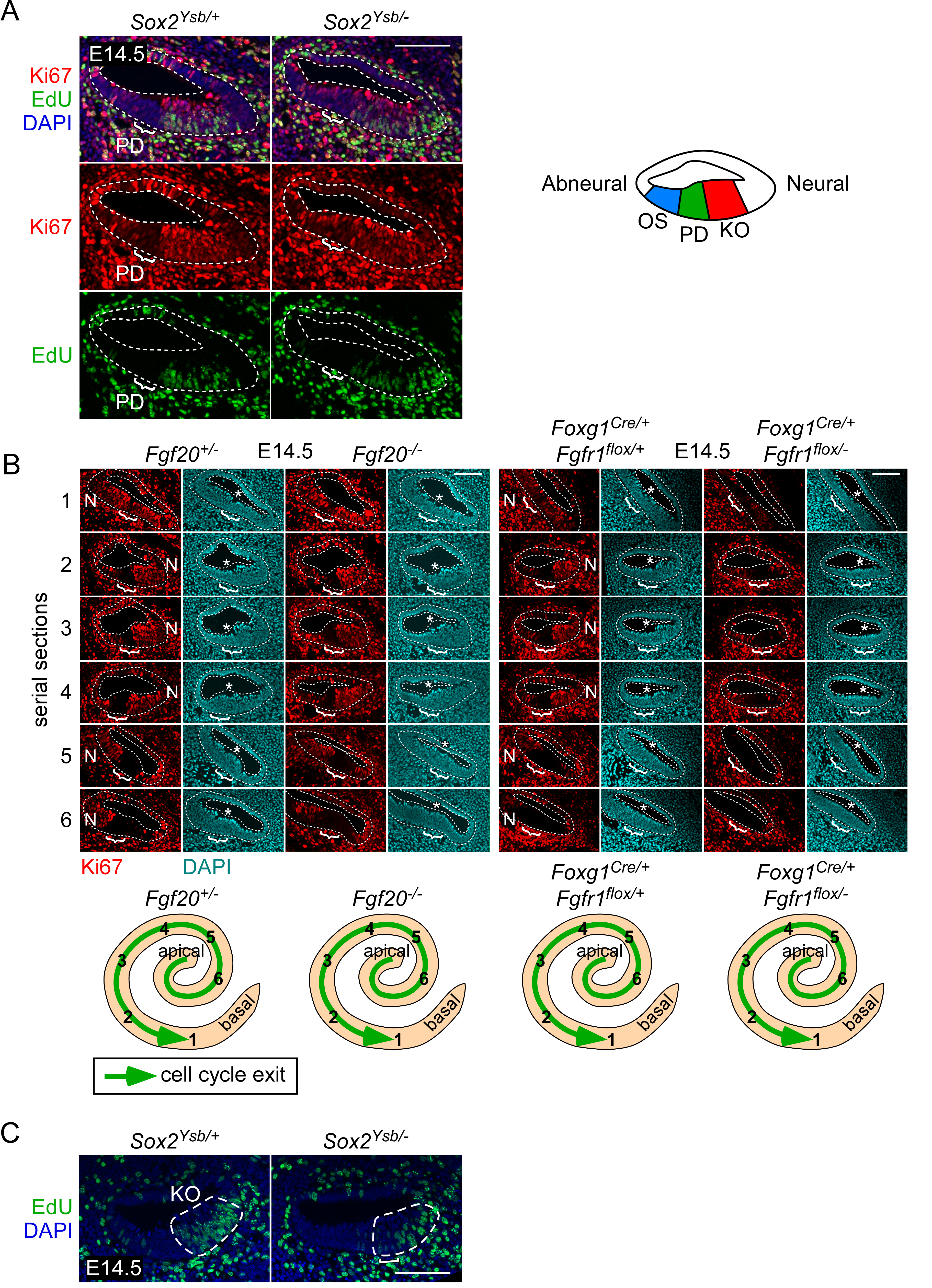

Supplement: S6 Fig — (A) Immunofluorescence for Ki67 (red) and EdU-incorporation (green) in sections through the basal turn of E14.5 Sox2Ysb/+ and Sox2Ysb/- cochleae. Cochlear epithelium is outlined. Bracket indicates the prosensory domain (PD). Samples are representative of n = 3, 3. (B) Serial sections (1–6) through the duct of E14.5 Fgf20+/-, Fgf20-/-, and Foxg1Cre/+;Fgfr1flox/+, Foxg1Cre/+;Fgfr1flox/- cochleae. Immunofluorescence for Ki67 (red) and DAPI (nuclei, cyan). Cochlear epithelium is outlined. Bracket indicates prosensory domain. * indicates shift of prosensory nuclei away from the luminal surface of the epithelium. N, neural side. Samples are representative of n = 6, 6, 5, 5. Whole mount cochlear duct schematics show relative positions of the serial sections and progression of cell cycle exit (green arrow). (C) EdU-incorporation (green) in sections through the middle turn of E14.5 Sox2Ysb/+ and Sox2Ysb/- cochleae. Dashed region indicates Kölliker’s organ (KO). Bracket indicates part of Kölliker’s organ without EdU-incorporating cells in Sox2Ysb/- cochleae. Samples are representative of n = 3, 3. OS, outer sulcus; PD, prosensory domain; KO, Kölliker’s organ. DAPI, nuclei (blue). Scale bar, 100 μm. (TIF) [file pgen.1008254.s006.tif]

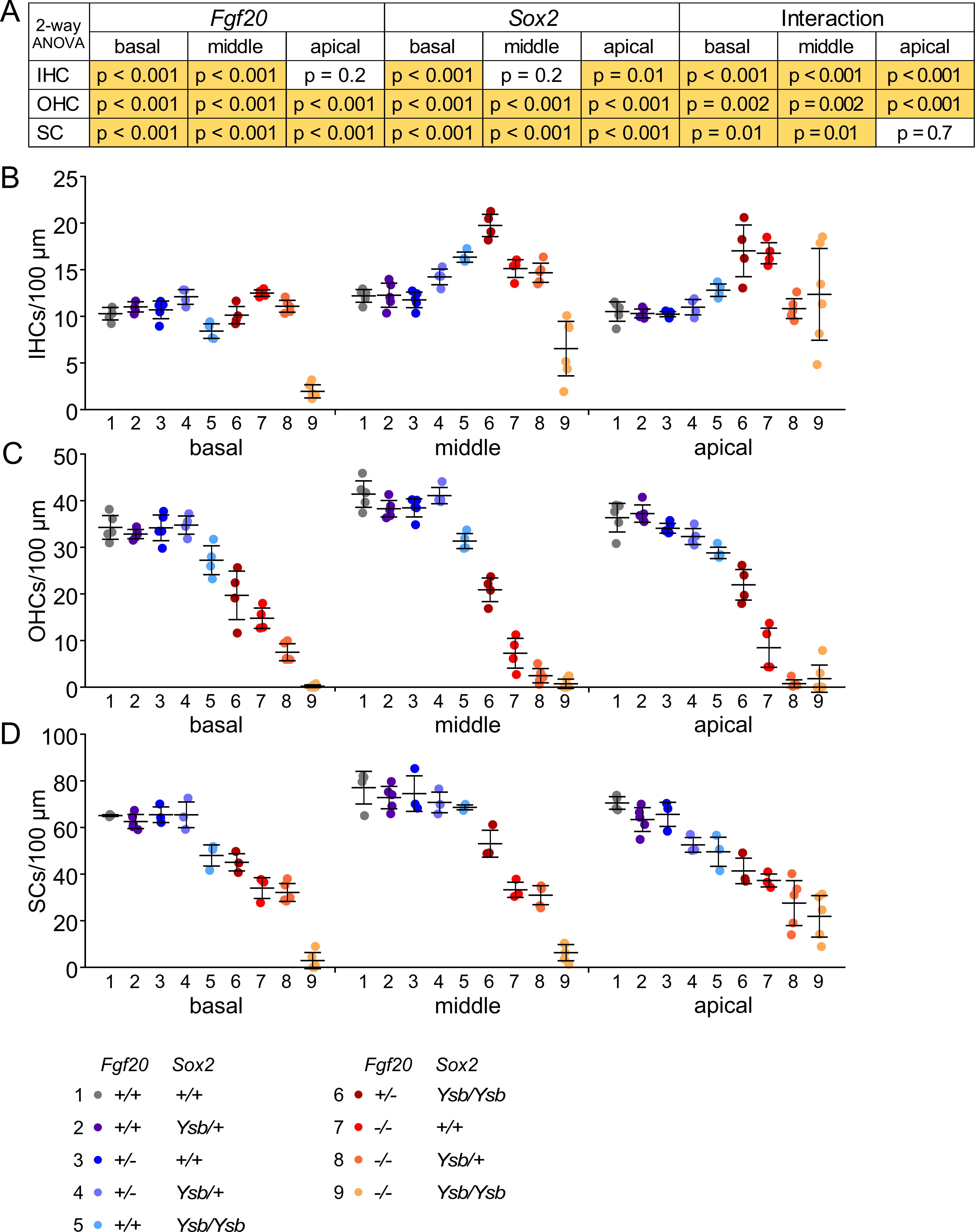

Supplement: S7 Fig — (A) P values from two-way ANOVA analyzing the quantification in (B-D). The two factors analyzed are Fgf20 (Fgf20+/+, Fgf20+/-, Fgf20-/-) and Sox2 (Sox2+/+, Sox2Ysb/+, Sox2Ysb/Ysb) gene dosage. A p value < 0.05 (yellow highlight) for Fgf20 or Sox2 indicates that the particular factor (independent variable) has a statistically significant effect on the measurement (dependent variable). Whereas a p value < 0.05 for Interaction indicates a statistically significant interaction between the effects of the two factors on the measurement. (B-D) Quantification of length-normalized number of (B) inner hair cells (IHCs/100 μm), (C) outer hair cells (OHCs/100 μm), and (D) supporting cells (SCs/100 μm) in the basal, middle, and apical turns of P0 cochleae from Fgf20+/+;Sox2+/+, Fgf20+/+;Sox2Ysb/+, Fgf20+/-;Sox2+/+, Fgf20+/-;Sox2Ysb/+, Fgf20+/+;Sox2Ysb/Ysb, Fgf20+/-;Sox2Ysb/Ysb, Fgf20-/-;Sox2+/+, Fgf20-/-;Sox2Ysb/+, and Fgf20-/-;Sox2Ysb/Ysb mice. Error bars, mean ± SD. n = (B, C) 5, 5, 5, 4, 4, 4, 4, 5, 6; (D) 4, 5, 3, 3, 3, 3, 3, 5, 5. (TIF) [file pgen.1008254.s007.tif]

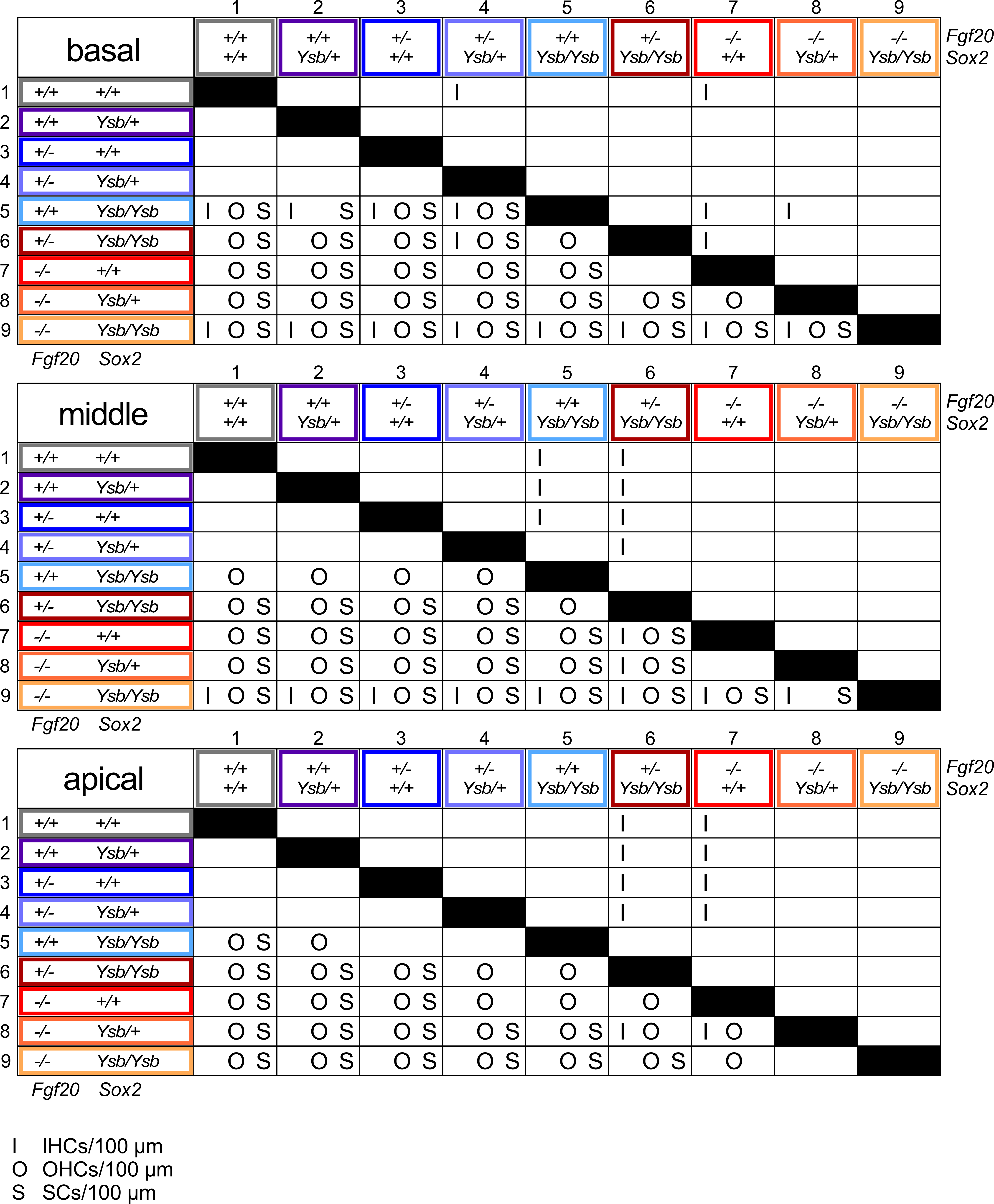

Supplement: S8 Fig — Results from post-hoc Tukey’s HSD analyzing the quantification results in (B-D). Letters (I, O, S; representing each measurement in S7B–S7D Fig) indicate a statistically significant decrease (p < 0.05) when comparing the row genotype against the column genotype. L, cochlear length; I, IHCs/100 μm; O, OHCs/100 μm; S, SCs/100 μm. (TIF) [file pgen.1008254.s008.tif]

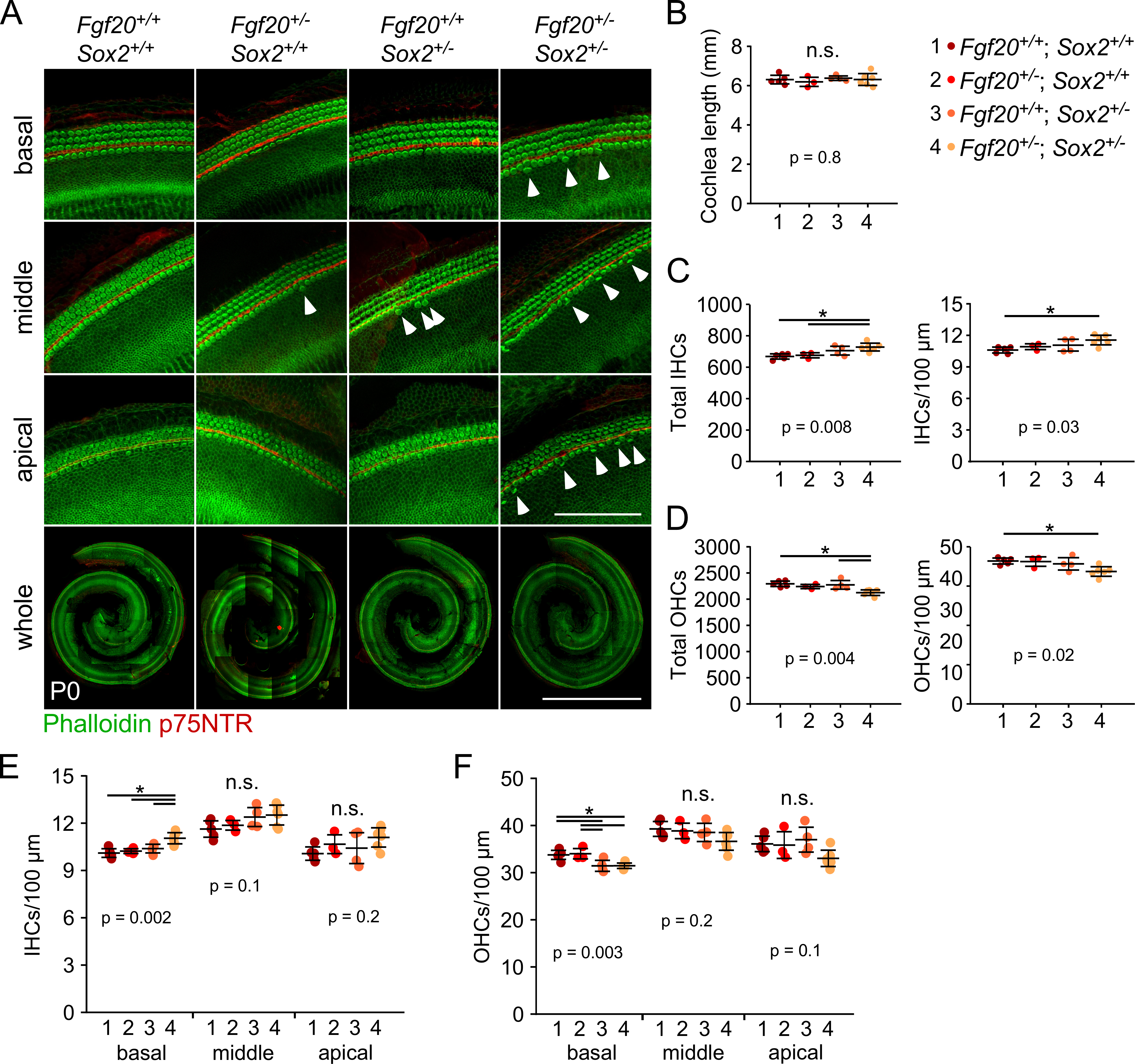

Supplement: S9 Fig — (A) Whole mount cochlea from P0 Fgf20+/+;Sox2+/+, Fgf20+/-;Sox2+/+, Fgf20+/+;Sox2+/-, and Fgf20+/-;Sox2+/- mice showing inner and outer hair cells (phalloidin, green) separated by inner pillar cells (p75NTR, red). Magnifications show the basal, middle, and apical turns of the cochlea. Scale bar, 100 μm (magnifications), 1 mm (whole); arrowheads indicate ectopic inner hair cells. (B-F) Quantification of (B) cochlear duct length, (C) total inner hair cells (IHCs) and IHCs per 100 μm of the cochlear duct, (D) total outer hair cells (OHCs) and OHCs per 100 μm, and (E) IHCs/100 μm and (F) OHCs/100 μm in the basal, middle, and apical turns at P0. P values shown are from one-way ANOVA. * indicates p < 0.05 from Tukey’s HSD (ANOVA post-hoc); n.s., not significant. Error bars, mean ± SD. n = 5, 3, 4, 6. (TIF) [file pgen.1008254.s009.tif]

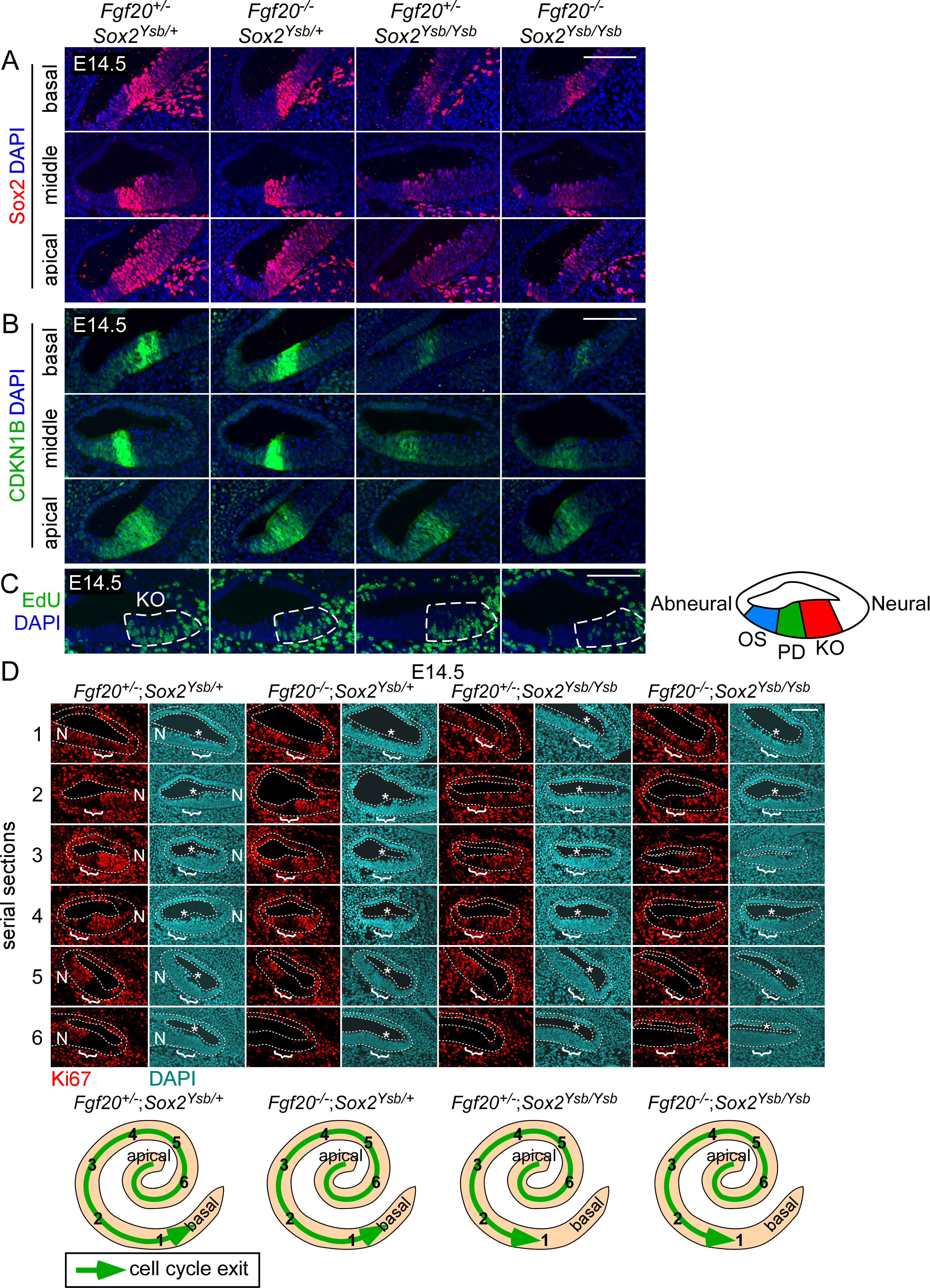

Supplement: S10 Fig — (A, B) Immunofluorescence for (A) Sox2 (red) and (B) CDKN1B (green) in sections through the basal, middle, and apical turns of E14.5 Fgf20+/-;Sox2Ysb/+, Fgf20-/-;Sox2Ysb/+, Fgf20+/-;Sox2Ysb/Ysb, and Fgf20-/-;Sox2Ysb/Ysb cochleae. Samples are representative of n = (A) 4, 4, 4, 4; (B) 5, 5, 5, 5. (C) EdU-incorporation (green) in sections through the middle turn of E14.5 Fgf20+/-;Sox2Ysb/+, Fgf20-/-;Sox2Ysb/+, Fgf20+/-;Sox2Ysb/Ysb, and Fgf20-/-;Sox2Ysb/Ysb cochleae. Dashed region indicates Kölliker’s organ (KO). Samples are representative of n = 3, 3, 3, 3. (D) Serial sections (1–6) through the duct of E14.5 Fgf20+/-;Sox2Ysb/+, Fgf20+/-;Sox2Ysb/Ysb, Fgf20-/-;Sox2Ysb/+, and Fgf20-/-;Sox2Ysb/Ysb cochleae. Immunofluorescence for Ki67 (red) and DAPI (nuclei, cyan). Cochlear epithelium is outlined. Bracket indicates prosensory domain. * indicates shift of prosensory nuclei away from the luminal surface of the epithelium. N, neural side. Samples are representative of n = 4, 4, 4, 4. Whole mount cochlear duct schematics show relative positions of the serial sections and progression of cell cycle exit (green arrow). Note: unlike in Fig 7, the placement of images from Fgf20-/-;Sox2Ysb/+ and Fgf20+/-;Sox2Ysb/Ysb cochleae have been switched to facilitate comparison. OS, outer sulcus; PD, prosensory domain; KO, Kölliker’s organ. DAPI, nuclei (blue). Scale bar, 100 μm. (TIF) [file pgen.1008254.s010.tif]
